# Supplementary material for: Long-term variations of arterial stiffness in patients with obesity and obstructive sleep apnea treated with continuous positive airway pressure
Source: PLoS One. 2020 Aug 5;15(8):e0236667. doi: 10.1371/journal.pone.0236667 (PMC7406029; doi:10.1371/journal.pone.0236667)
Supplement: S1 Table — AHI, apnea hypopnea index; HbA1c, Glycated hemoglobin; hs-CRP, high sensitivity C-reactive protein; DBP, diastolic blood pressure; FEV1, Forced Expiratory Volume of the first second of forced expiration; FVC, Forced Vital Capacity; SBP, systolic blood pressure; SpO2, oxygen saturation; TLC, total lung capacity. (DOCX) [file pone.0236667.s001.docx]

**S1 Table. Comparison between imputed and non-imputed datasets**

| Variable | Non-imputed data | Imputed data | Missing values (N) | P |
| --- | --- | --- | --- | --- |
| Smoking | 40 (59.7) | 43 (59.7) | 5 | 1.00 |
| SBP (mmHg) | 132 [122 ; 140] | 132 [122.5 ; 139.5] | 2 | 0.99 |
| DBP (mmHg) | 79.5 [70 ; 85] | 79.5 [69 ; 85] | 2 | 0.96 |
| HbA1c | 5.8 [5.5 ; 6.3] | 5.8 [5.5 ; 6.3] | 3 | 0.98 |
| Fasting Glucose (mmol/l) | 5.7 [5.3 ; 6.2] | 5.7 [5.3 ; 6.2] | 6 | 0.98 |
| Insulinemia (µUl/ml) | 8.7 [6.4 ; 13.3] | 8.7 [6.4 ; 13.3] | 8 | 0.99 |
| hsCRP (mg/l) | 4.2 [2.1 ; 8.9] | 4.4 [2.1 ; 9.2] | 8 | 0.79 |
| FVC (% of predicted value) | 99 [84 ; 106] | 97.5 [84 ; 106] | 3 | 0.87 |
| EV1 (% of predicted value) | 92 [82 ; 103] | 91 [82 ; 102.5] | 3 | 0.81 |
| FEV1/ FVC (%) | 80.6 [75.5 ; 84.1] | 80.2 [74.3 ; 84] | 3 | 0.84 |
| VFEV1/ FVC < 70% | 7 (10.1) | 7 (9.7) | 3 | 0.93 |
| TLC (% of predicted value) | 103.5 [96.5 ; 114] | 104 [97 ; 113] | 8 | 0.97 |
| Epworth Sleepiness Scale | 12 [8 ; 16] | 12 [8 ; 16] | 1 | 0.97 |
| AHI (/hour) | 36.1 [23.3 ; 75.2] | 37.6 [24.8 ; 74.6] | 2 | 0.98 |
| Mean nocturnal SpO2 (% of total sleep time) | 92 [89 ; 94] | 92 [89 ; 94] | 2 | 0.99 |
| Sleep time spent with SpO2 < 90% (% of total sleep time) | 11 [2 ; 43] | 11.1 [2.2 ; 43] | 3 | 0.90 |

There were no differences between imputed and non-imputed datasets for variables with missing values.
