# Supplementary material for: Long-term variations of arterial stiffness in patients with obesity and obstructive sleep apnea treated with continuous positive airway pressure
Source: PLoS One. 2020 Aug 5;15(8):e0236667. doi: 10.1371/journal.pone.0236667 (PMC7406029; doi:10.1371/journal.pone.0236667)
Supplement: S2 Table — (DOCX) [file pone.0236667.s002.docx]

**S2 Table. Medication used by patients at the second assessment**

| Antihypertensives   - monotherapy - bitherapy - tritherapy - quadritherapy | 44%  12.5%  9.7%  5,6% |
| --- | --- |
| Oral anti-diabetic drugs | 30.6% |
| Insulin | 11.1% |
| Lipid-lowering drugs | 34.7% |
| Antiplatelet drugs | 18.1% |
| Anticoagulants | 4.2% |
| long acting bronchodilators | 15.3% |
| Corticosteroids | 1.4% |
| Non-steroidal anti-inflammatory drugs | 2.8% |
| Anti-secretory therapies | 30.6% |
| Anti-allergics | 15.3% |
| Gout medications | 13.9% |
| Thyroid hormones | 18.1% |
| Opiate analgesics | 13.9% |
| Antidepressants | 19.4% |
| Antiparkinson therapies | 2.8% |
| Hypnotics / Anxiolytics | 13.9% |
| Antiepileptic drugs | 2.8% |
| Neuroleptic drugs | 2.8% |

Proportion of patients using each therapeutic
