# Supplementary material for: Long-term variations of arterial stiffness in patients with obesity and obstructive sleep apnea treated with continuous positive airway pressure
Source: PLoS One. 2020 Aug 5;15(8):e0236667. doi: 10.1371/journal.pone.0236667 (PMC7406029; doi:10.1371/journal.pone.0236667)
Supplement: Attachment — Submitted filename: Response to Reviewers 7-7-20.docx [file pone.0236667.s006.docx]

**Response To Reviewers**

PONE-D-20-05576R1

Long-term variations of arterial stiffness in patients with obesity and obstructive sleep apnea treated with continuous positive airway pressure

*Thank you to the reviewers for their responses and comments.*

*The responses to the reviewers are made in subsection 6 of the document.*

Reviewers' comments:

Reviewer's Responses to Questions

**Comments to the Author**

1. If the authors have adequately addressed your comments raised in a previous round of review and you feel that this manuscript is now acceptable for publication, you may indicate that here to bypass the “Comments to the Author” section, enter your conflict of interest statement in the “Confidential to Editor” section, and submit your "Accept" recommendation.

Reviewer #1: All comments have been addressed

Reviewer #2: (No Response)

2. Is the manuscript technically sound, and do the data support the conclusions?

The manuscript must describe a technically sound piece of scientific research with data that supports the conclusions. Experiments must have been conducted rigorously, with appropriate controls, replication, and sample sizes. The conclusions must be drawn appropriately based on the data presented. 

Reviewer #1: Yes

Reviewer #2: Yes

3. Has the statistical analysis been performed appropriately and rigorously? 

Reviewer #1: Yes

Reviewer #2: Yes

4. Have the authors made all data underlying the findings in their manuscript fully available?

The [PLOS Data policy](http://www.plosone.org/static/policies.action#sharing) requires authors to make all data underlying the findings described in their manuscript fully available without restriction, with rare exception (please refer to the Data Availability Statement in the manuscript PDF file). The data should be provided as part of the manuscript or its supporting information, or deposited to a public repository. For example, in addition to summary statistics, the data points behind means, medians and variance measures should be available. If there are restrictions on publicly sharing data—e.g. participant privacy or use of data from a third party—those must be specified.

Reviewer #1: Yes

Reviewer #2: Yes

5. Is the manuscript presented in an intelligible fashion and written in standard English?

PLOS ONE does not copyedit accepted manuscripts, so the language in submitted articles must be clear, correct, and unambiguous. Any typographical or grammatical errors should be corrected at revision, so please note any specific errors here.

Reviewer #1: Yes

Reviewer #2: Yes

6. Review Comments to the Author

Please use the space provided to explain your answers to the questions above. You may also include additional comments for the author, including concerns about dual publication, research ethics, or publication ethics. (Please upload your review as an attachment if it exceeds 20,000 characters)

Reviewer #1: I would like to thank the authors for revising the manuscript. I think it substantially improved. I have no further comments/questions.

Reviewer #2: Authors have addressed the major part of our recommendations.
But some few corrections need to be done:
Line 153: the reference of AASM need to be in the reference list *: The reference has been added in the text*
Line 178: if “FEV1/FVC<70% was one pre-specified inclusion criteria” you should write it in the section participant as an inclusion criteria. Also, if you write according to standard definitions, we need to have a reference. *: FEV1 / FVC <70% was not an inclusion criteria of our study. We use FEV1 / FVC <70% to define the presence of airflow limitation on pulmonary function test. A reference to FEV1 / FVC <70% defining the airflow limitation has been added in the text.*
Line 180-183: “We thank the reviewer for asking for clarification on this. CPAP adherence used for data analysis was corresponding to objective compliance measured in the 3 to 6 months preceding follow-up visit. This sentence has been added in the methods section.” I am not seeing that sentence in the section on CPAP treatment *: Indeed, this sentence did not appear in the method section. The sentence "CPAP adherence used for data analysis was corresponding to objective compliance measured in the 3 to 6 months preceding follow-up visit" was added in the CPAP treatment subsection of the method section.*
Line 243: delete “figure 3” in the title of the section “multivariate analysis” and put it in the text below *: Done*
Line 311: please, in the conclusion it is not appropriate to write details as “1.34 [0.10; 2.37] m/s” … etc. You can write: “there is an increase in PWV over the study period. Determinants of PWV progression are old age and hypertension” *: Thank you for your comment. The change has been made in the text.*

7. PLOS authors have the option to publish the peer review history of their article ([what does this mean?](https://journals.plos.org/plosone/s/editorial-and-peer-review-process#loc-peer-review-history)). If published, this will include your full peer review and any attached files.


If you choose “no”, your identity will remain anonymous but your review may still be made public.


**Do you want your identity to be public for this peer review?** For information about this choice, including consent withdrawal, please see our [Privacy Policy](https://www.plos.org/privacy-policy).

Reviewer #1: No

Reviewer #2: Yes: Virginie Poka-Mayap
